# Epidemiological Investigation of OHCWs with COVID-19

**DOI:** 10.1177/0022034520962087

**Published:** 2020-09-27

**Authors:** L. Meng, B. Ma, Y. Cheng, Z. Bian

**Affiliations:** 1 The State Key Laboratory Breeding Base of Basic Science of Stomatology (Hubei-MOST) & Key Laboratory of Oral Biomedicine Ministry of Education, School & Hospital of Stomatology, Wuhan University, Wuhan, Hubei, China; 2 Center for Evidence-Based Stomatology, School & Hospital of Stomatology, Wuhan University, Wuhan, Hubei, China

**Keywords:** dental public health, virology, infection control, patient outcomes, dental education, infectious disease

## Abstract

During the coronavirus disease 2019 (COVID-19) pandemic, there is an important risk of infection in health care workers, including oral health care workers (OHCWs), due to the characteristics of dental practice. In this retrospective study, data pertaining to the 31 OHCWs diagnosed with COVID-19 in Wuhan, Hubei Province, were retrieved and analyzed. Questionnaires were administered to the subjects over the Internet and telephone. Clinical and epidemiological characteristics and information on the use of personal protective equipment (PPE) were collected. There were 22 females and 9 males, with a median age of 39 y. Although the severity of most cases of COVID-19 (93.5%) was mild or moderate, 1 case was severe, and another case was critical, resulting in death. Fever was the main first symptom of COVID-19, followed by fatigue and myalgia. Most of the OHCWs routinely used PPE such as medical masks, gloves, caps, and work clothes while performing clinical work. N95 or KN95 masks were rarely available because of the scarcity of PPE during the outbreak. Nineteen OHCWs reported a contact history, among whom 7 worked in a fever clinic, 5 reported contact with dental patients suspected of having COVID-19, and 7 reported contact with family members with COVID-19–related symptoms at least 1 d earlier. Our findings indicated that there were few clusters of COVID-19 in dental settings. Since the outbreak, the Hospital of Stomatology, Wuhan University, has provided emergency dental treatment, and none of their staff were infected while providing dental service, which indicates that comprehensive measures such as the use of advanced PPE and environmental disinfection can prevent cross-infection in dental practice. The analysis of the procedures followed during the emergency treatments indicated that OHCWs preferred to recommend conservative treatment to patients, suggesting that attention should be paid to the psychological impact of COVID-19 on dental practitioners.

## Introduction

As of August 31, 2020, the sustained coronavirus disease 2019 (COVID-19) pandemic had resulted in nearly 25 million laboratory-confirmed cases and 800,000 deaths worldwide (World Health Organization 2020). This disease is caused by severe acute respiratory syndrome coronavirus 2 (SARS-CoV-2). Its human-to-human transmission occurs mainly via respiratory droplets and direct contact, although it may also occur via the fecal-oral route or aerosols (National Health Commission of China [NHC] 2020a).

In an analysis of 138 hospitalized COVID-19 patients in Zhongnan Hospital in Wuhan, China, 57 patients were presumed to have been infected in the hospital, including 40 health care workers (HCWs) (Wang et al. 2020). In a retrospective analysis of 1,099 patients with confirmed COVID-19 in 551 hospitals from 30 provinces, autonomous regions, and municipalities across China, the proportion of those infected who were HCWs was 3.5% (Guan et al. 2020). As of February 11, 2020, 1,716 health care personnel had contracted COVID-19, representing 3.8% of the affected individuals nationally, and 5 had died (Wu and McGoogan 2020). In the Lombardy area of Italy, the proportion of infected individuals who were HCWs was 20% (*n* = 350), and some died (Remuzzi and Remuzzi 2020). As of April 9, 2020, a total of 9,282 HCWs in the United States had confirmed COVID-19 according to the report from the Centers for Disease Control and Prevention (CDC COVID-19 Response Team 2020). These data indicate that HCWs are at risk of infection.

Close contact, the spatter of oral secretions, saliva, or blood, and aerosols generated by high-speed handpieces or ultrasonic instruments create a presumed risk of nosocomial infection in dental practice (Dave et al. 2020; Peng et al. 2020). A recent article reported that 9 oral health care workers (OHCWs) from the Hospital of Stomatology, Wuhan University had confirmed COVID-19 (Meng et al. 2020). However, further information on the infection of OHCWs with SARS-CoV-2, such as their basic demographic characteristics, the disease severity distribution, and a transmission analysis, is still lacking in the literature. Hence, we retrospectively investigated the epidemiological and clinical characteristics of 31 OHCWs with COVID-19 in Wuhan and the prevention and control measures in their workplaces. Such an analysis of infected OHCWs may help identify their clinical characteristics and aid in the development of sustainable oral health care services as the economy and nonessential medical services reopen in affected areas.

## Methods

### Ethics Approval

This retrospective study was approved by the ethics committee of the Hospital and School of Stomatology, Wuhan University (No. 2020-B29). Informed consent was obtained from the included subjects through express delivery.

### Data Sources

To obtain information about the infected OHCWs, we initially retrieved data from the Hubei Stomatological Association (HSA) between January 1 and March 31, 2020. There are 3,566 HSA members in Hubei Province, of whom 1,533 are in Wuhan, including 394 members in private dental clinics. Thereafter, to confirm the data, we made phone calls to all the related dental institutions in Wuhan. Information on 32 infected OHCWs in Wuhan was reported. Then, we further determined whether there were infected OHCWs in the other 34 public general or specialized hospitals based on the data from the Health Annals of Wuhan in 2019. Information regarding another 2 OHCWs with COVID-19 in Wuhan was also collected. With regard to the private dental clinics, 183 dental clinics in Wuhan employ approximately 606 OHCWs. However, a regulation was released by the Hubei government on January 27, 2020, regarding dental services during the outbreak that stated that only emergency dental cases could be treated during the strict implementation of infection prevention and control measures. All private clinics were closed in Wuhan during the epidemic, and the information on infection was mainly obtained from the situation report released by the HSA, which reported 4 infected OHCWs who worked in 3 private clinics in Wuhan.

Both telephone interviews and questionnaires were used to collect information from the OHCWs with COVID-19. We developed a questionnaire (Table 1) to collect the epidemiological and clinical information, which consisted of 3 parts: 1) general information: name, age, gender, name of medical institution, occupation, and department; 2) clinical information: clinical symptoms and signs, admission and discharge dates, and medical discharge record; and 3) epidemiological information: exposure history, direct work in a fever clinic, potential source of infection, infection status in occupational or household settings, and prevention and control measures in the workplace.

**Table 1.** Questionnaire of Oral Health Care Workers with COVID-19 in Wuhan.

**Part 1**. General information Name: Birthday: yyyy-mm-dd Address: Gender: Name of medical institution: Occupation and department: **Part 2**. Clinical information 1. Onset of symptoms and time: yyyy-mm-dd □ Fever □ Cough □ Sputum production □ Hemoptysis □ Conjunctival congestion □ Fatigue □ Chest distress □ Sore throat □ Headache □ Myalgia □ Arthralgia □ Chills □ Diarrhea □ Nausea or vomiting □ Dyspnea □ Others 2. Time for confirmation: yyyy-mm-dd 3. Signs and symptoms (Please ✓): □ Fever □ Cough □ Sputum production □ Hemoptysis □ Conjunctival congestion □ Fatigue □ Chest distress □ Sore throat □ Headache □ Myalgia □ Arthralgia □ Chills □ Diarrhea □ Nausea or vomiting □ Dyspnea □ Others 4. Coexisting disorders (Please ✓): □ Hypertension □ Coronary heart disease □ Cerebrovascular diseases □ Diabetes □ Chronic pulmonary diseases □ Chronic renal diseases □ Chronic liver disease □ Immunodeficiency diseases □ Caner □ Other □ None 5. Hospitalization (Please ✓): □ Yes, yyyy-mm-dd □ No 6. Discharge (Please ✓): □ Yes, yyyy-mm-dd □ No 7. Relapse (Please ✓): □ Yes, yyyy-mm-dd □ No **Part 3**. Epidemiologic information 1. Contact history with confirmed or suspected cases of COVID-19 (Please ✓): □ Yes (If yes, please describe time of exposure and/or onset of symptom) □ Family member, yyyy-mm-dd (□ couple □ parent □ child □ other) □ Colleague, yyyy-mm-dd (□ in your department □ the other department) □ Patient, yyyy-mm-dd □ Other, specify: yyyy-mm-dd □ No 2. Whether working in fever clinic before onset of symptom? (Please ✓): □ Yes, yyyy-mm-dd □ No 3. Is there any confirmed or suspected case in your family or department? □ Yes (If yes, please describe time of exposure or onset of symptom) □ Family member, yyyy-mm-dd □ Colleague yyyy-mm-dd □ No 4. Select your prevention and control measures in the workplace (Please ✓): □ Disposable medical masks □ Caps □ Disposable gloves □ N95 or KN95 masks □ Facial shields □ Goggles □ Work clothes □ Gowns □ Protective clothes □ Hand hygiene □ Other □ None 5. Time off duty: yyyy-mm-dd

To collect information on dental services performed during the epidemic, the medical records pertaining to emergency dental treatments were collected from the Hospital of Stomatology, Wuhan University as an example from January 23 to April 7, 2020. Diagnosis and treatments were recorded and classified.

### Statistical Analysis

Continuous variables are expressed as the medians and interquartile ranges. Categorical variables are summarized as counts and percentages. Because the subjects in our study were not randomly selected, all statistics are purely descriptive. All the analyses were performed with IBM SPSS Statistics 19.0 (SPSS, Inc.).

## Results

### Epidemiologic and Clinical Characteristics

A total of 34 OHCWs diagnosed with COVID-19 were identified in Wuhan. Among them, 30 OHCWs and a family member of a deceased OHCW gave informed consent, and 3 declined to be interviewed. The clinical information of the deceased dentist was provided by his family members, one of whom was also one of the aforementioned 30 OHCWs.

All the subjects were categorized into patients who were positive for SAR-CoV-2 nucleic acid (confirmed cases) and those who had been diagnosed based on clinical manifestations and pathological chest radiology or computed tomography (CT) imaging (diagnosed cases). Patients were also divided into groups based on whether they had had mild, moderate, severe, and critical COVID-19 according to the latest COVID-19 guidelines from the NHC (2020a).

The 31 OHCWs included in this study were from 13 general hospitals, 1 Hospital of Stomatology, 1 community health service center, and 3 private clinics. There were 22 females (71.0%) and 9 males (29.0%) (Table 2). The median age of the subjects was 39 y (interquartile range, 32 to 49). Of them, 96.8% were 20 to 59 y, and only 1 was over 60 y. Regarding their occupations, 19 (61.3%) were dentists, 10 (32.3%) were nurses, and 2 (6.4%) were administrators.

**Table 2.** General Demographic Characteristics of Oral Health Care Workers Diagnosed with COVID-19 in Wuhan.

Demographic Characteristics	Categories	No. (%)
Gender	Male	9 (29.0)
	Female	22 (71.0)
Age	20 to 39	17 (54.8)
	40 to 59	13 (41.9)
	≥60	1 (3.2)
Occupation	Dentist	19 (61.3)
	Nurse	10 (32.3)
	Administrator	2 (6.4)
Diagnosis	Confirmed	27 (87.1)
	Clinical diagnosed	4 (12.9)
Signs and symptoms	Fever	27 (87.1)
	Fatigue	13 (41.9)
	Myalgia	12 (38.7)
	Cough	11 (35.5)
	Diarrhea	7 (22.6)
	Chest distress	6 (19.4)
	Chills	6 (19.4)
Severity of disease	Mild or moderate	29 (93.6)
	Severe	1 (3.2)
	Critical	1 (3.2)
Coexisting disorders	Hypertension	2 (6.4)
	Diabetes	2 (6.4)
	Chronic renal diseases	1 (3.2)
	Tuberculosis	1 (3.2)
	Cancer	1 (3.2)
	Hepatitis B infection	1 (3.2)
	None	25 (80.6)

**Figure 1.**
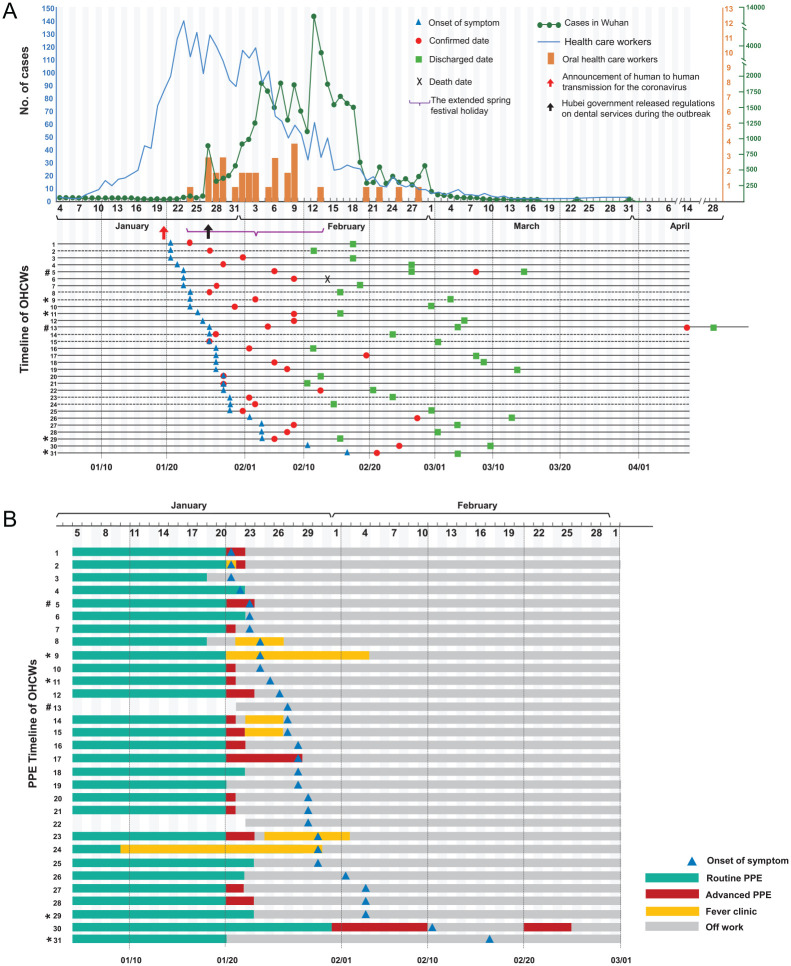
The epidemiological characteristics and personal protective equipment (PPE) usage of the infected oral health care workers (OHCWs). (**A**) Number of confirmed cases of coronavirus disease 2019 (COVID-19) in Wuhan (NHC 2020c), confirmed cases in health care workers in Wuhan (China Red Cross Foundation 2020), and infected OHCWs since the outbreak (from January 1 to March 31, 2020) according to the date of confirmation. Above the date line, the orange bars represent the number of the OHCWs confirmed or diagnosed every day. Below the date line, the date of the earliest onset of symptoms, confirmation or diagnosed date, discharge date, and death date are labeled for each subject, as appropriate. The dashed lines represent 7 OHCWs who worked in a fever clinic and were later confirmed to have COVID-19. (**B**) PPE usage of the infected OHCWs. Twenty-one of the OHCWs (67.7%) reported working in the department of stomatology or the dental clinic and using routine PPE before January 20, 2020. Twelve of the 21 OHCWs gradually started to use advanced PPE, including disposable surgical masks, goggles or face shields, and gowns, although N95 or KN95 masks were rarely available. Nos. 13 and 22 were 2 administrators who did not use PPE. Seven OHCWs were assigned to the fever clinic and used variable PPE to protect themselves from contracting COVID-19, including surgical masks, N95 or KN95 masks, caps, gloves, and gowns or protective clothes. An OHCW (No. 30) was assigned to the surgical ward during the epidemic and did not improve her PPE until February 1. She had COVID-19–related symptoms on February 11 and returned to work on February 21 after symptom improvement and 2 consecutive negative chest computed tomography (CT) examinations. She was confirmed as having COVID-19 on February 25 when nucleic acid tests became available in her hospital. *OHCWs (Nos. 9, 11, 29, 31), who had clinical manifestations of infection and pathological changes in chest CT but negative nucleic acid test results, were diagnosed with COVID-19 according to the guideline of clinical diagnosis of COVID-19 (NHC 2020b). The guidelines added the clinical diagnosis to the classification of COVID-19 in Hubei Province. ^#^Two OHCWs (Nos. 5 and 13) whose reverse transcriptase polymerase chain reaction tests for severe acute respiratory syndrome coronavirus 2 became positive again after discharge. They were discharged the second time on March 15 and April 28, 2020.

Twenty-seven patients had positive results on a real-time reverse transcriptase polymerase chain reaction (RT-PCR) assay of nasal or pharyngeal swab specimens, while the other 4 patients were diagnosed based on their clinical presentation and chest radiology or CT (NHC 2020b). Fever (87.1%) was the main first symptom of COVID-19, followed by fatigue (41.9%) and myalgia (38.7%). The earliest onset of symptoms in 29 of the 31 patients occurred between January 21 and February 4, 2020 (Fig. 1A). The median interval between the onset of symptoms and diagnosis or confirmation was 6 d (interquartile range, 3 to 14). Twenty-nine patients were categorized for severity of disease as mild or moderate types (93.6%), 1 as a severe type, and 1 as a critical type who died. In addition, 25 (80.6%) of the OHCWs reported no coexisting disorders, but the 2 patients with severe and critical COVID-19 had breast cancer with hypertension and type 2 diabetes, respectively.

Of the 31 OHCWs, 1 died, and the remaining 30 met the criteria for discharge in China (absence of clinical symptoms and radiological abnormalities, with 2 consecutive negative RT-PCR results) and were released from the hospital. The individual who unfortunately died was male and aged 40 y with type 2 diabetes. His initial symptoms were cough and fever on January 23, 2020. He was hospitalized 6 d later and then admitted to the intensive care unit because of his worsening condition. His diagnosis was confirmed on February 9, and he died on February 14. Two of 30 OHCWs had positive RT-PCR test results again 10 and 40 d after being discharged. Of these, 1 was a dentist, and the other was an administrator. Both recovered and were discharged again (Nos. 5, 13; Fig. 1).

### Prevention and Control Measures Taken by the OHCWs

Of the 31 OHCWs, 21 (67.7%) reported working in the department of stomatology or the dental clinic and using routine personal protective equipment (PPE), including work clothes, caps, disposable medical masks, and gloves, before January 20, 2020. Because of the official announcement of human-to-human transmission of SARS-CoV-2 on January 20, 12 of the 21 OHCWs started gradually using advanced PPE, including disposable surgical masks, goggles or facial shields, and gowns. N95 or KN95 masks were rarely available because of the scarcity of PPE in the early stage of the epidemic.

There were 2 administrators who did not routinely use PPE (Nos. 13 and 22; Fig. 1B). Seven OHCWs were assigned to the fever clinic that was first introduced during the SARS epidemic in 2003 and were involved in the triage and management of fever patients. They used a variety of PPE to protect from COVID-19, including surgical masks, N95 or KN95 masks, caps, gloves, and gowns or protective clothes, based on the regulations regarding prevention and control measures and the PPE available in different hospitals. They worked in the fever clinic from 1 d to almost 3 wk. One OHCW (No. 30) was assigned to the surgical ward during the epidemic and did not improve her PPE until February 1.

Most of the OHCWs had not treated patients since January 24, 2020, because of the extended Chinese Spring Festival from January 24 to February 13 and the regulation released by the Hubei government on January 27 regarding the provision of dental services during the outbreak. Only 1 OHCW worked in the dental emergency department (No. 17), 1 in a surgical ward (No. 30), and 6 in the fever clinic until the onset of symptoms occurred or COVID-19 was confirmed.

### Possible Route of Infection

**Figure 2.**
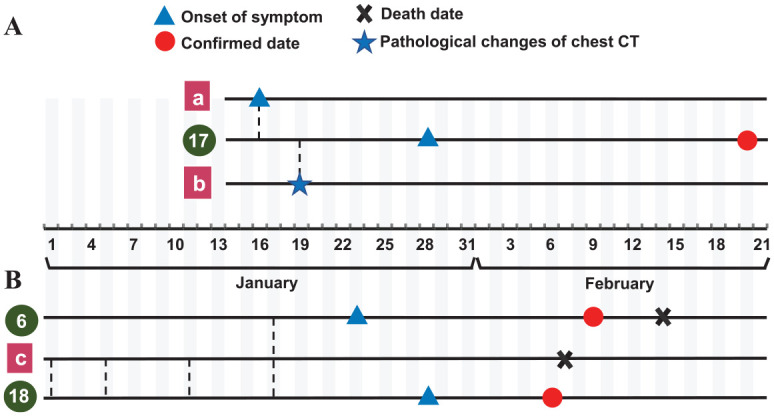
Timetable of 3 oral health care workers (OHCWs) with potential nosocomial transmission in dental settings. The dashed lines represent the dates on which the OHCWs treated or had contact with dental patients with suspected or confirmed coronavirus disease 2019 (COVID-19). (**A**) One OHCW (No. 17) worked in the department of stomatology of a general hospital. The dentist reported treating a patient (a) with root canal obturation with the use of a high-speed handpiece on January 16. The patient (a) had a fever later that day. He had pathological manifestations on chest computed tomography (CT) on January 17 and then received confirmation of infection at the end of January. On January 19, the dentist treated another patient (b) and performed the second stage of an implant surgery. The patient (b) was diagnosed with suspected COVID-19 based on chest CT that day and received confirmation later. These 2 dental patients were No. 17’s colleagues from other departments in the hospital. On January 28, the dentist developed a fever and received confirmation on February 20. His assistant, a 28-y-old male resident, had negative result on both the nucleic acid test and serum immunoglobulin (IgG/IgM) test. The dentist treated 30 patients from January 16 to 28. Except for 2 confirmed patients, 6 of them reported being healthy during follow-up, and the others were lost to follow-up. (**B**) The OHCWs (Nos. 6 and 18) were siblings. They worked at the same clinic but did not live together. No. 18 reported treating a dental patient (c) 4 times, including performing root canal treatment and periodontal treatment. No. 6 had a close conversation with the patient without wearing a medical mask on January 17. The patient (c) was a 77-y-old woman with a history of hyperuricemia. Her date of symptom onset was unknown. She had a fever and typical chest CT manifestation of COVID-19, and she died on February 7 without having received a nucleic acid test. The 2 dentists treated 92 patients in January 2020. During follow-up, 65 dental patients or their families answered the phone and 64 reported being healthy, while a family member reported that 1 patient had died (c).

Among these OHCWs, 16 (51.6%) worked in the department of stomatology of hospitals designated for the treatment of COVID-19 and/or hospitals that had fever clinics. Within 14 d prior to disease onset, 19 (61.3%) of the OHCWs reported contact with confirmed or suspected cases of COVID-19 in either health care or household settings. Seven (22.6%) were assigned to work in the fever clinic because of the shortage of the health care workforce during the epidemic, and later, they developed COVID-19–related symptoms. Five (16.1%) reported exposure history in dental settings. One of them (No. 17) treated 2 dental patients who received confirmation of COVID-19 before he did (Fig. 2A). These 2 dental patients (a, b) were colleagues of the OHCW (No. 17) and worked in other departments in a general hospital. The dentist treated 30 dental patients from January 16 to 28. Aside from the 2 patients with confirmed COVID-19, 6 patients reported being healthy during follow-up, and the others were lost to follow-up. As shown in Figure 2B, an OHCW (No. 18) reported treating a dental patient (c) who was suspected of having COVID-19. The treatments included root canal treatment and periodontal treatment. Her brother, who was a dentist (No. 6), engaged in a close conversation with that patient while not wearing a medical mask on January 17. In total, these 2 dentists treated 92 patients in January 2020. During the follow-up, 65 dental patients or their families answered the phone and 64 reported being in good health, while 1 family member reported that the patient had died (c). Another 2 OHCWs (Nos. 12 and No. 28) reported either having contact with a febrile patient or treating a dental patient who had been in close contact with COVID-19 patients on January 23. However, the information regarding the patients was not available or traceable. None of the other close relatives or colleagues of these OHCWs (Nos. 6, 12, 17, 18, 28) had confirmed COVID-19.

The family members of 7 (22.6%) OHCWs exhibited COVID-19 symptoms at least 1 d earlier before the OHCWs experienced symptom onset. The remaining 12 subjects reported no clear contact history, of whom 3 had family members with confirmed COVID-19 but with the onset of symptoms later than themselves.

Six OHCWs with COVID-19 worked in 3 different departments other than the fever clinic (Nos. 6 and 18, 16 and 27, 11 and 28 in 3 departments or clinics) (Fig. 1), which cannot exclude possible transmission of infection in dental settings.

### Dental Emergency Treatment during the Outbreak

**Figure 3.**
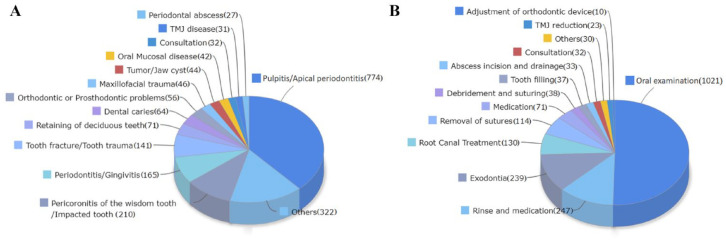
Classification of cases during emergency dental treatments in the Hospital of Stomatology, Wuhan University from January 23 to April 7, 2020. (**A**) Classification according to diagnosis. Others include follow-up after treatment, stitch removal, loss of a filling, complications after implant restoration, and so on. (**B**) Classification according to dental procedures. Others included tooth fixation, tooth replantation, removal of foreign matter, reattachment of a fractured tooth, and so on.

The Hospital of Stomatology, Wuhan University has provided dental emergency treatment during the outbreak. In total, 320 staff served 2,025 patients with increasingly advanced PPE, including N95 or KN95 masks, and equipment and environment disinfection measures from January 23 to April 7, 2020, and none of them were infected. As shown in Figure 3, 67.3% of the treated patients had pulpitis/apical periodontitis (774), pericoronitis of the wisdom tooth/impacted tooth (210), periodontitis/gingivitis (165), tooth fracture/tooth trauma (141), maxillofacial trauma (46), and periodontal abscess (27) related to toothache and trauma. As for operations, oral examinations (1,021) accounted for nearly half, followed by rinse and medication (247), exodontia (239), and root canal treatment (130), which accounted for approximately 30.4%.

## Discussion

### OHCWs with COVID-19

Our study analyzed the 31 COVID-19 OHCWs in Wuhan from January 1 to March 31 and found that 22 of the 31 patients (71%) were female, which is not consistent with the results of previous studies about COVID-19 in the general population or HCWs (Chu et al. 2020; Guan et al. 2020; Huang et al. 2020; Li et al. 2020; Pan et al. 2020; Zhang 2020). It may be explained that the majority of OHCWs are female (Ayers et al. 2008; Kfouri et al. 2019). Two recent online reports about HCWs with COVID-19 showed that in China and the United States, more than 70% of HCWs were female (Bytedance Health Care Worker Humanitarian Relief Fund 2020; CDC COVID-19 Response Team 2020), which is in agreement with our results.

The main symptoms among OHCWs with COVID-19 were fever, fatigue, and myalgia. Their manifestations are basically consistent with those in other confirmed patients in Wuhan, most of whom had relatively mild symptoms (Guan et al. 2020; Zhang 2020). The proportion of mild or moderate cases among the OHCWs (93.6%) was higher than that among the HCWs (20% to 85%) and the general population (78% to 84%) (Chu et al. 2020; Guan et al. 2020; Pan et al. 2020; Wu and McGoogan 2020; Zhang 2020). Although the specific reasons for the difference are unknown, it is possible that younger age, fewer coexisting diseases, or a higher proportion of female patients in the present study influenced the disease severity (Guan et al. 2020; Li et al. 2020; Pan et al. 2020; Wu and McGoogan 2020; Yang et al. 2020).

There were 4 medical staff in Wuhan and 25 patients in Shenzhen who had positive PCR results again after recovering from COVID-19 (Lan et al. 2020; Yuan et al. 2020). Two of the OHCWs in the study, one of whom was a dentist and the other of whom was an administrator, had similar experiences. The “recurrence” might be related to the biological characteristics of SARS-CoV-2, the patient’s coexisting disorder, clinical status, sampling, or other factors (Zhou et al. 2020). Although recovered patients may continue to shed detectable SARS-CoV-2 RNA, they are unlikely to be contagious 10 to 20 d after onset of symptoms as the live virus can no longer be cultured (CDC 2020). Therefore, a symptom-based strategy for determining when HCWs with COVID-19 can return to work has been recommended (CDC 2020). There is no consensus about when or how convalescent COVID-19 patients can be treated in dental clinics. Further study is needed to determine the clinical and pathological characteristics of the disease.

Due to the characteristics of dental procedures producing splatters and aerosols and the transmission mode of SARS-CoV-2, it is presumed there is a very high risk of infection for OHCWs. However, this seemed to not be the case in this study. In an analysis of COVID-19 HCWs in China, the most high-risk occupations were in the respiratory and emergency department, while the department of stomatology was not included in the top 10 (Bytedance Health Care Worker Humanitarian Relief Fund 2020). This may be related to dental practitioners’ emphasis on hand hygiene, equipment and environmental disinfection, and routine PPE usage in dental practices even prior to the outbreak of COVID-19. During the SARS epidemic, a case-control study showed that proper use of standard precautions was adequate to prevent the nosocomial spread of SARS in the absence of aerosol-producing procedures (Samaranayake and Peiris 2004). Moreover, our 320 staff served 2,025 dental emergency patients during the epidemic, and none of them were infected, further indicating that comprehensive measures such as the use of advanced PPE and equipment and environmental disinfection are effective at preventing cross-infection in dental setting. Another possible explanation is that dental patients are less likely to be acutely ill than inpatients. Therefore, the likelihood of encountering an infected person is reduced in a dental setting. In addition, the outbreak of COVID-19 occurred during the extended Chinese Spring Festival holiday from January 24 to February 13, 2020, and the Hubei government released the regulation limiting dental services during the outbreak on January 27. Routine dental practices have been suspended until further notice. These restrictions reduced the influx of patients into the hospital and decreased the potential risk of cross-infection (Samaranayake and Peiris 2004; Meng et al. 2020).

In our study, 19 (61.3%) OHCWs reported contact with patients with confirmed or suspected COVID-19 in either health care or household settings. Twelve (38.7%) OHCWs reported the contact occurring in health care settings, which was similar to the exposure reported by HCWs in the United States (CDC COVID-19 Response Team 2020). The human-to-human transmission of the virus occurs during close contact with an infected person by exposure to respiratory droplets expelled by coughing, sneezing, or aerosols (Dave et al. 2020; Peng et al. 2020; Shereen et al. 2020). In the study, 5 OHCWs reported having been exposed in dental settings. Moreover, 3 different departments had more than 1 OHCW with COVID-19, highlighting the possible transmission of infection in dental settings. The possible route of infection in this study was based on the report from the OHCWs and their families. Case investigation and contact tracing are the most effective methods of establishing the route of virus transmission. However, they alone possibly fail to identify the origin of a case of COVID-19. Genomic sequencing of SARS-CoV-2 clarifies the probable source of infection in cases in which epidemiological links could not be determined (Rockett et al. 2020). Recently, complete genome sequences of virus strains from patients in a family cluster and clusters of HCWs unveiled plausible transmission pathways when combined with detailed clinical and epidemiological data (Chan et al. 2020; Rivett et al. 2020). However, it is expensive for OHCWs to travel to offsite testing facilities, and it was impossible to collect virus samples in this retrospective study because 30 of the OHCWs had already been discharged and 1 had died.

Several studies have reported that COVID-19 occurs in family clusters (Chan et al. 2020; Qian et al. 2020). The family members of 7 OHCWs exhibited COVID-19–related symptoms or were diagnosed before the OHCWs. Another 3 OHCWs had family members with confirmed cases of COVID-19, but the OHCWs experienced the onset of symptoms first. These findings all indicate the possibility of intrafamilial infection.

The possible routes of infection highlight several important points about prevention. Many medical staff were not adequately protected and therefore became infected (Guan et al. 2020; Remuzzi and Remuzzi 2020; Wang et al. 2020; Wu and McGoogan 2020). N95 masks or protective clothes were rarely available for the OHCWs in this study because of the scarcity of PPE during the COVID-19 pandemic. HCWs should be provided with sufficient protective equipment to prevent viral transmission in health care settings. Adequate training (Chu et al. 2020) and monitoring for compliance with standard precautions are also important. Regular COVID-19 testing of HCWs, including OHCWs at a high risk for personal or occupational reasons, may be valuable (CDC COVID-19 Response Team 2020; Dave et al. 2020; El-Boghdadly et al. 2020; Rivett et al. 2020).

### Data on Patient Treatment

Dental procedures generating droplets and aerosols, especially when patients are in the incubation period, increase the risk of transmission among dental practitioners and patients (Harrel et al. 2004; Meng et al. 2020; Peng et al. 2020). During an outbreak, HCWs became increasingly reluctant to work due to the long hours of working under pressure and the risk of infection (Schwartz et al. 2020). Multiple factors that can influence whether dentists are willing to treat patients may include PPE availability, clinical environment, patient attitudes, and ethics consideration of regarding the patients’ right to receive treatment. Although dental practitioners should have the primary goal of relieving the patient’s pain, dentists may fear transmission among colleagues, patients, and family members (The Lancet 2020). From our data on emergency dental treatment, for example, there were 774 cases of pulpitis and periapical periodontitis, most of which should have been treated routinely in the dental emergency department such as via root canal treatment. However, only 130 patients received this procedure, indicating that the OHCWs preferred to recommend conservative treatment. A recent study reported that most dentists (82.6%) preferred to avoid working with a patient with a suspected case of COVID-19, and approximately half (43.8%) mentioned that they would refer the patient to the hospital without treating them during the outbreak of COVID-19 (Khader et al. 2020). These results all reflect the psychological impact of COVID-19 on OHCWs. It is critical to strengthen OHCWs’ sense of safety as well as their trust in their workplace.

### Strengths and Limitations

This study is based on retrospective data, which might have led to recall bias to a certain extent. The exposures might have occurred earlier than those reported in the retrospective data. Therefore, another limitation is the inability to conclude if these were truly household or health care exposures. Although we tried to contact all the departments and private clinics in Wuhan, we were unable to reach some private clinics because of the epidemic. We may also have missed asymptomatic OHCWs or those who had mild symptoms and were treated at home. Thus, our study may not be fully representative of all OHCWs with COVID-19 in Wuhan.

## Conclusion

During the outbreak of COVID-19, there was a high risk of infection in dental settings, as expected. Therefore, it is imperative to employ strict personal protective measures and to delay nonemergency dental treatment due to an increased risk of community transmission and/or a strain on the health care system such as PPE preserved for frontline workers. However, there is also a need to provide routine and preventive health care when reasonable to preserve oral health and diagnose unrecognized illness. We should balance the provision of dental services and the reduction in the risk of infection in dental settings. In addition, attention should be paid to the psychological impact of COVID-19 on OHCWs, which may be critical to provide sustainable oral health care services and reopen the dental clinics after the pandemic.

## Author Contributions

L. Meng, Z. Bian, contributed to conception, design, data acquisition, analysis, and interpretation, drafted and critically revised the manuscript; B. Ma, Y. Cheng, contributed to design, data acquisition, and analysis. All authors gave final approval and agree to be accountable for all aspects of the work.
